# Medulloblastoma in the cerebellopontine angle mimicking a schwannoma

**DOI:** 10.1002/ccr3.3912

**Published:** 2021-02-12

**Authors:** Le Thanh Dung, Nguyen Minh Duc

**Affiliations:** ^1^ Department of Radiology Viet Duc Hospital Ha Noi Vietnam; ^2^ Department of Radiology Hanoi Medical University Ha Noi Vietnam; ^3^ Department of Radiology Pham Ngoc Thach University of Medicine Ho Chi Minh City Vietnam; ^4^ Department of Radiology Children's Hospital 2 Ho Chi Minh City Vietnam

**Keywords:** cerebellopontine angle, children, extra‐axial, medulloblastoma

## Abstract

The typical distinction between cerebellopontine angle (CPA) medulloblastoma and other primary CPA tumors was not fully known. While CPA medulloblastoma is very uncommon, it should be included in the differential diagnosis of patients with CPA tumors.

## Graphical abstract

Medulloblastoma is a highly malignant brain tumor that most often occurs in children. These tumors are usually located in the vermis or fourth ventricle. Extra‐axial cerebellopontine angle (CPA) medulloblastoma is exceptionally rare. We describe a case of CPA medulloblastoma mimicking a schwannoma in a 6‐year‐old girl. Although the patient underwent many innovative magnetic resonance imaging (MRI) sequences, the corrected diagnosis was not initially established. The typical discrimination between CPA medulloblastoma and other primary CPA tumors was not fully clarified. This paper underscores that while CPA medulloblastoma is exceedingly rare, it should still be considered in the differential diagnosis of patients with CPA tumors.

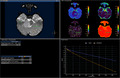

## 1 INTRODUCTION

Medulloblastoma is an aggressive and profoundly malignant neuroectodermal tumor of the cerebellum that accounts for around 10% of all pediatric brain neoplasms.^1^ Approximately, 75% of medulloblastomas emerge from the fourth ventricle and vermis in children, while most medulloblastomas arise superiorly from the cerebellar hemispheres in adults.^2^ Since medulloblastoma is primarily intra‐axial, exclusively extra‐axial medulloblastomas at the cerebellopontine angle (CPA) are quite rare. The overwhelming majority of CPA tumors are cholesteatoma, epidermoid, meningioma, or schwannoma.^3^ In this article, we describe a rare case of CPA medulloblastoma in a 6‐year‐old girl, which was originally misdiagnosed as schwannoma. We highlight that accurate diagnosis of CPA medulloblastoma remains problematic, despite advances in magnetic resonance imaging (MRI) protocol.

## 2 CASE PRESENTATION

**FIGURE 1:**
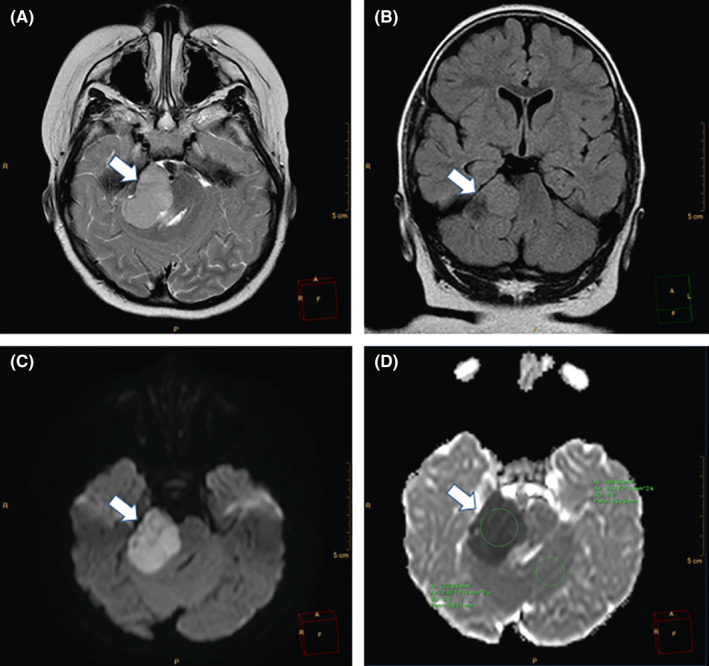
A homogeneously hyperintense solid mass located in the right CPA, on axial T2‐weighted image (arrow, A). The mass appears isointense in coronal FLAIR imaging (arrow, B), hyperintense on axial DWI imaging (arrow, C), and hypointense on the axial ADC map (arrow, D)

**FIGURE 2:**
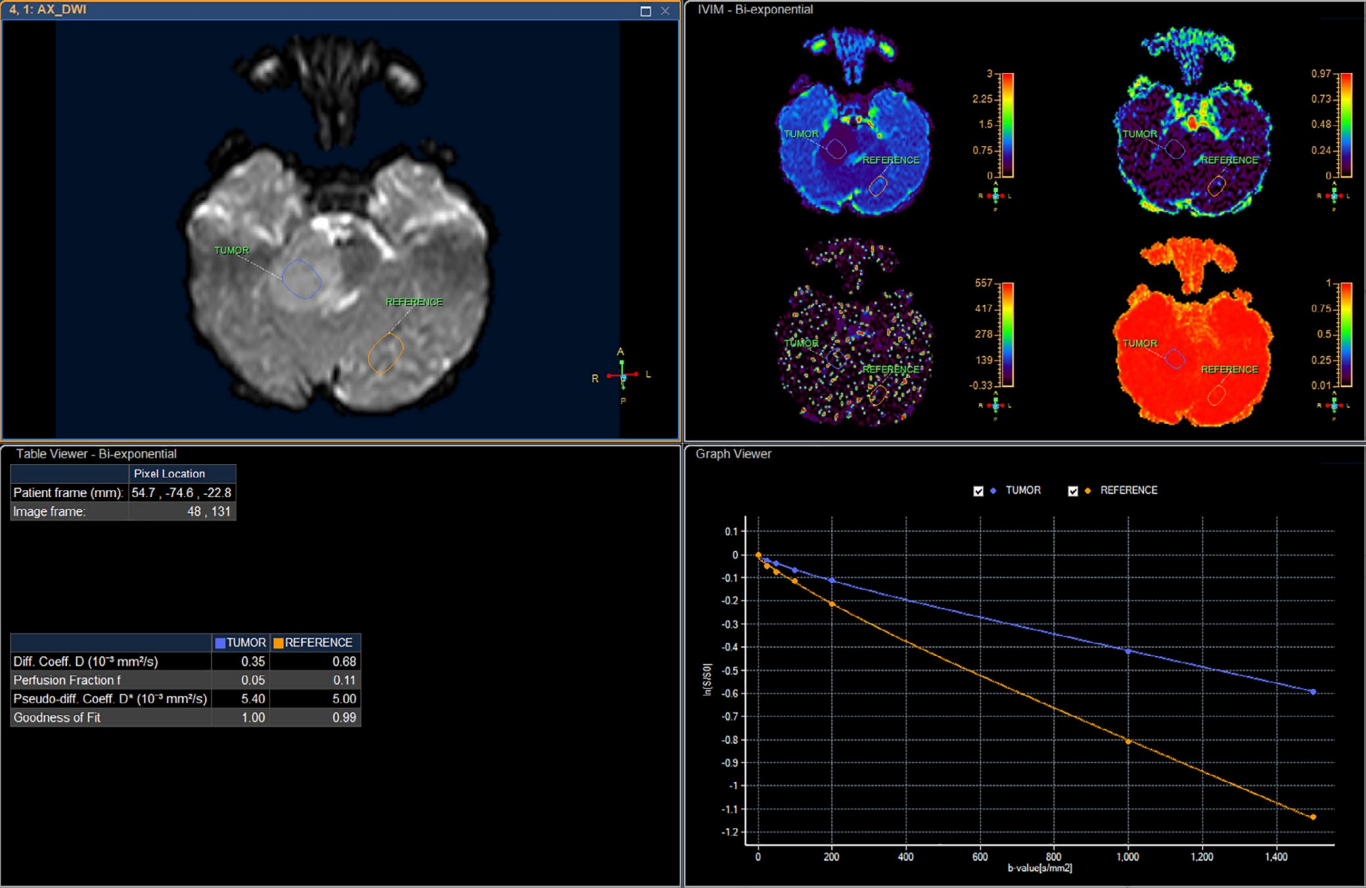
Intravoxel incoherent motion analysis for the tumor and parenchyma

**FIGURE 3:**
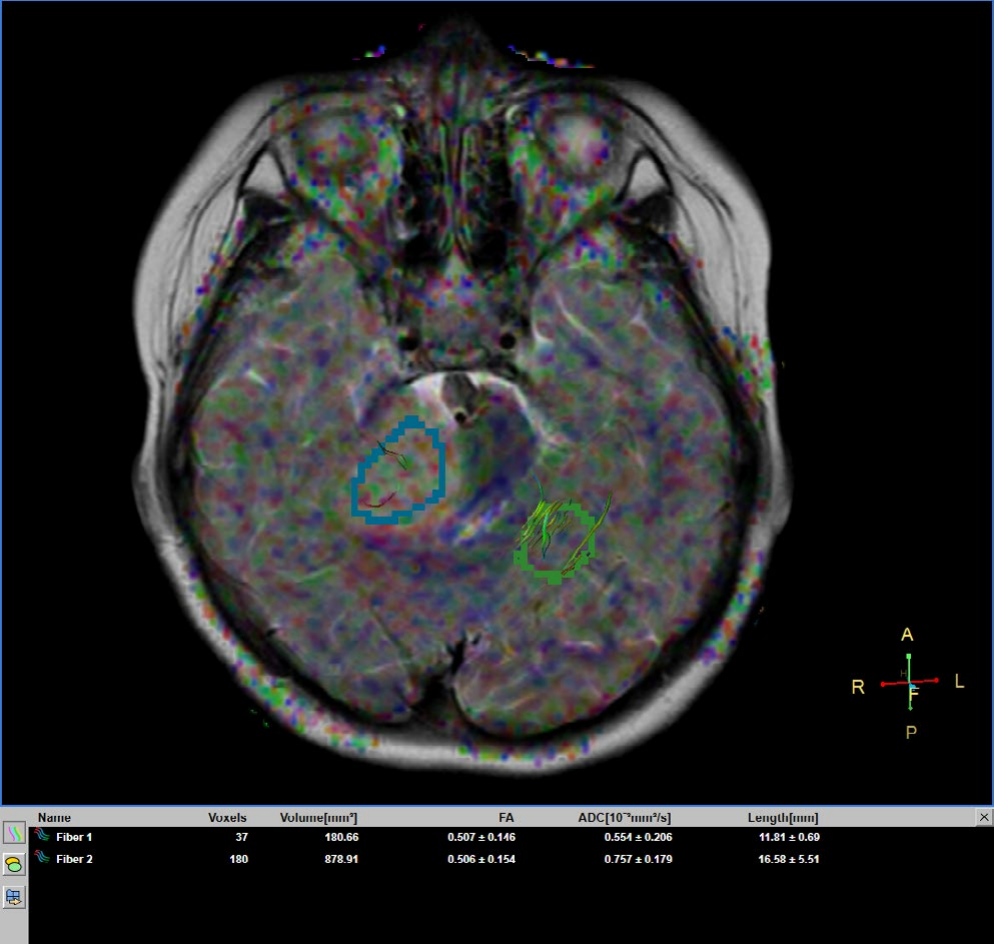
The FA values for the tumor and the parenchyma on diffusion tensor imaging

**FIGURE 4:**
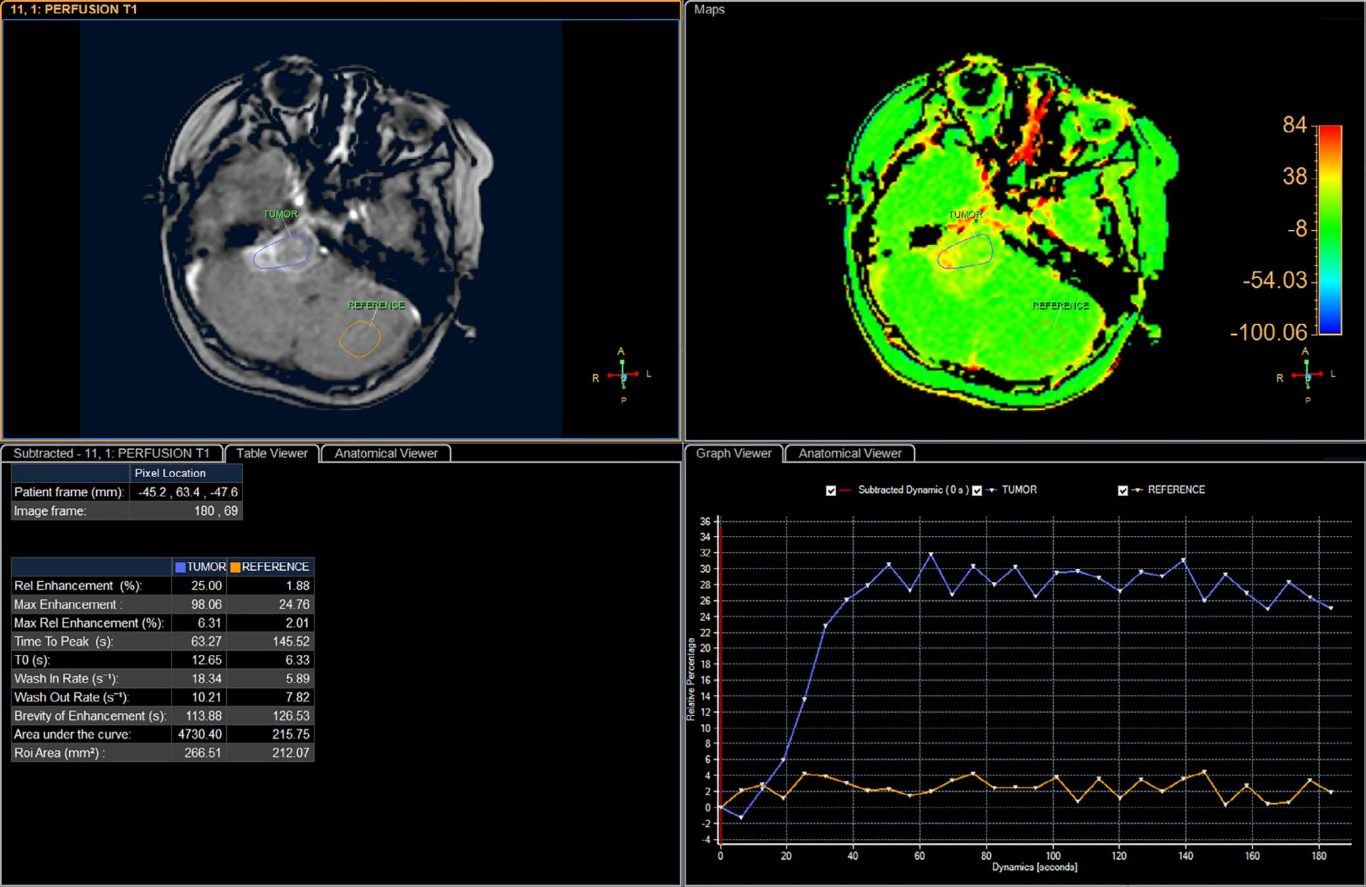
Perfusion analysis for the tumor and the parenchyma on the semiquantitative T1‐perfusion map

**FIGURE 5:**
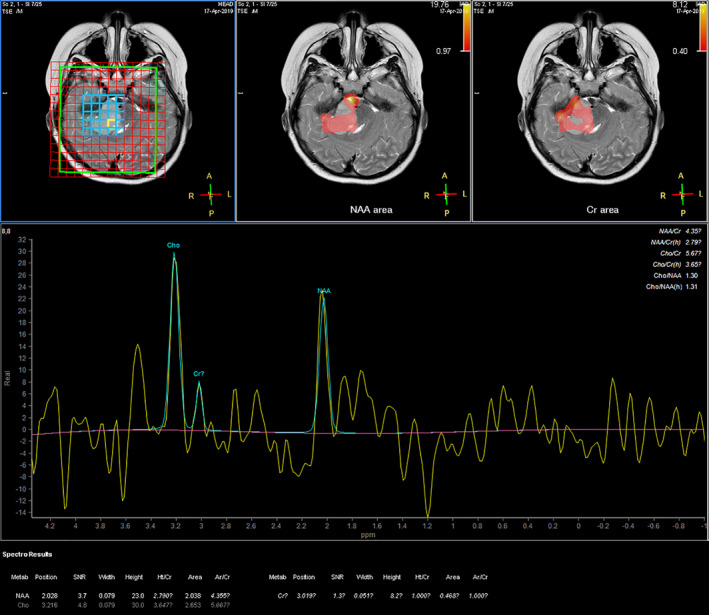
Metabolic parameters of the tumor based on MRI spectroscopy

**FIGURE 6:**
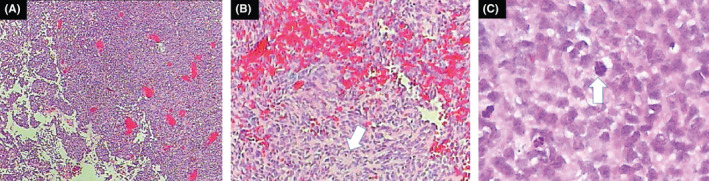
Histopathological results showing (A) densely packed small‐blue‐round cells and (B) embryonal cells with Homer Wright rosettes, accompanied by (C) high mitotic activity (hematoxylin and eosin staining, original magnification [A], ×100 [B], and ×400 [C])

A 6‐year‐old girl suffering from seizure and hearing loss was admitted to Children's Hospital 2 in Ho Chi Minh City, Vietnam. The patient's medical profile was normal. Upon clinical assessment, no neurological defects were observed. Laboratory test findings were within normal ranges. The patient underwent brain magnetic resonance imaging (MRI) with a gadolinium‐based contrast agent. A homogeneous solid mass, situated in the right CPA, showed high signal intensity on T2‐weighted imaging (Figure 1A), appeared isointense on fluid‐attenuated inversion recovery (FLAIR) imaging (Figure 1B), appeared hyperintense on diffusion‐weighted imaging (DWI, Figure 1C), and showed low signal intensity on apparent diffusion coefficient imaging (ADC, Figure 1D) images. The size of mass was 27 mm × 38 mm × 34 mm. There were no signs of ossification, edema, or hemorrhage related to the mass. The mean ADC values for the mass and normal‐appearing parenchyma were 0.40 and 0.62 × 10^‐3^ mm^2^/s, respectively. The diffusivity (10^‐3^ mm^2^/s), perfusion fraction (%), and pseudo‐diffusivity (10^‐3^ mm^2^/s) values determined from intravoxel incoherent motion (IVIM) analysis were and were 0.35, 0.05, 5.40 for the tumor and 0.68, 0.11, 5.00 for the normal‐appearing parenchyma (Figure 2). The fractional anisotropy (FA) values calculated from diffusion tensor imaging (DTI) were 0.507 for the mass and 0.506 for the normal‐appearing parenchyma (Figure 3). The relative enhancement (%), maximum enhancement, maximum relative enhancement (%), time to peak (s), wash‐in rate (s^‐1^), wash‐out rate (s^‐1^), and area under the curve values derived from the axial T1‐perfusion map for the tumor compared with that of the parenchyma were 25.00 vs 1.88, 98.06 vs 24.76, 6.31 vs 2.01, 63.27 vs 145.52, 18.34 vs 5.89, 10.21 vs 7.82, and 4730.40 vs 215.75, respectively (Figure 4). The choline/N‐acetyl‐aspartate ratio for the mass was 1.30, as measured by MRI spectroscopy (Figure 5). Based on clinical and radiological data, the preliminary diagnosis was a schwannoma. However, after a radical dissection, the histopathological evaluation of the excised sample tissues was fully congruent with a classic medulloblastoma (Figure 6A‐C). The patient was uneventfully released 8 days postsurgery.

## 3 DISCUSSION

Medulloblastomas—extremely malignant embryonal tumors with a dismal prognosis—are classified as grade IV according to the WHO classification of central nervous system (CNS) tumors.^4^ Medulloblastomas are common pediatric intracranial tumors, accounting for about 40% of the posterior fossa tumors.^5^ Most medulloblastomas develop in the cerebellar midline at inferior vermis and typically expand into and occupy the fourth ventricle. A lower proportion of medulloblastomas are situated in the cerebellar hemispheres in adults.^6^, ^7^, ^8^, ^9^, ^10^, ^11^ Medulloblastomas usually induce intracranial hypertension and cerebellar dysfunction in both children and adults.^5^ Medulloblastomas are most commonly found extra‐axially in two areas: the tentorial region and CPA.^6^, ^7^, ^8^, ^9^, ^10^, ^11^

The origin of medulloblastomas in the CPA remains unclear. CPA medulloblastomas have been proposed to arise from residue in the external granular layer of the cerebellar hemisphere, specifically the flocculus facing the CPA. They can also emerge from the proliferation of the lateral medullary velum germinal residues, then protruding into the CPA. Medulloblastomas found in the fourth ventricle are often assumed to develop laterally to the CPA through the Luschka foramen or direct exophytic growth from the origin site at the surface of the cerebellum or pons.^12^, ^13^, ^14^

Unfortunately, CPA medulloblastoma may not have clear clinical and radiological characteristics that fully differentiate this neoplasm with other CPA tumors such as cholesteatoma, epidermoid, meningioma, and schwannoma.^3^, ^6^, ^7^, ^8^, ^9^, ^10^, ^11^ Furthermore, if a patient with CPA medulloblastoma experiences deafness, as was the case here, it is very difficult to distinguish CPA medulloblastoma from acoustic schwannoma. This is consistent with a prior report.^15^

The appearance of medulloblastoma as an extra‐axial mass also raises a preoperative diagnosis problem radiologically. Computed tomography (CT) reveals a well‐defined mass with heterogeneous contrast enhancement at CPA, while MRI shows a well‐defined mass with low signal intensity on T1‐weighted images and iso‐to‐high signal intensity on T2‐weighted images.^5^, ^16^ Due a high rate of cellular division and densely packed cells, the diffusion of the tumor is restricted and the tumor will manifest mostly as a homogeneous enhancement with the administration of gadolinium contrast.^5^, ^16^, ^17^, ^18^ In this case report, even though patient underwent advanced MRI sequences, the exact diagnosis of CPA medulloblastoma was dismissed since radiological information on CPA medulloblastoma and vestibular schwannoma was not understood thoroughly.

Four histological forms of medulloblastoma are recognized in the WHO 2007 Classification: classic medulloblastoma, desmoplastic/nodular medulloblastoma, large cell/anaplastic medulloblastoma, and medulloblastoma with extensive nodularity.^4^ A previous study reported that the most common variant of pediatric CPA medulloblastoma is classic medulloblastoma (43%).^10^

Radical neurosurgery is the first‐line treatment for CPA medulloblastoma. The combination of total gross resection with radiotherapy has been proven to enhance the long‐term prognosis. The 5‐year survival, just 30% in 1960s, has nearly doubled, owing to more innovative surgical techniques and effective adjuvant chemotherapies and radiotherapies.^19^

## 4 CONCLUSION

Cerebellopontine angle medulloblastomas are an outstandingly infrequent entity in children. The preoperative diagnosis is still troublesome due to the insufficiency of typical clinical and radiological features that discriminate CPA medulloblastoma from other CPA lesions such as cholesteatoma, epidermoid, meningioma, and schwannoma. Nonetheless, in the differential diagnosis of CPA tumors, CPA medulloblastoma should be taken into account in order to enhance the treatment plan and facilitate comprehensive post‐operative management.

## CONFLICT OF INTEREST

None declared.

## AUTHOR CONTRIBUTIONS

LTD and NMD: contributed equally to this work as co‐first authors. NMD: managed the patient. LTD and NMD: led data collection, conducted literature search, and wrote the initial draft of the manuscript. LTD and NMD: edited the draft into this manuscript. All authors approved the final version of the manuscript and agree to be accountable for all aspects of the work ensuring that questions related to the accuracy or integrity of any part of the work are appropriately investigated and resolved.

## ETHICAL APPROVAL

This study was approved by the Institutional review board of Children's Hospital 2 (Ref: 352/NĐ2‐CĐT). Written informed consent from the patient's legal guardian was obtained for the publication of this case report and any accompanying images.

## Data Availability

Data sharing is not applicable to this article as no datasets were generated or analyzed during the current study.
